# Supplementary material for: Computational 4D-OCM for label-free imaging of collective cell invasion and force-mediated deformations in collagen
Source: Sci Rep. 2021 Feb 2;11:2814. doi: 10.1038/s41598-021-81470-7 (PMC7854660; doi:10.1038/s41598-021-81470-7)
Supplement: Supplementary file 1 — Supplementary Information. [file 41598_2021_81470_MOESM1_ESM.pdf]

Supplementary Information for:

Computational 4D-OCM for label-free imaging of collective cell invasion and force-mediated deformations in collagen

Jeffrey A. Mulligan[1,2], Lu Ling[2], Nichaluk Leartprapun[2], Claudia Fischbach[2,3], Steven G. Adie[2,*]

[1] School of Electrical and Computer Engineering, Cornell University, Ithaca, NY, 14853 USA
[2] Nancy E. and Peter C. Meinig School of Biomedical Engineering, Cornell University, Ithaca, NY, 14853 USA
[3] Kavli Institute at Cornell for Nanoscale Science, Cornell University, Ithaca, NY, 14853 USA
[*] Corresponding author: sga42@cornell.edu

Contents:

Supplementary Note 1: Hardware-based compensation of coherence gate curvature (Model)

Supplementary Note 2: Hardware-based compensation of coherence gate curvature (Validation)

Supplementary Note 3: OCT image reconstruction procedure

Supplementary Note 4: Displacement tracking noise floor

Supplementary Note 5: Quantification of displacement tracking performance at varying spatial scales

Supplementary Note 6: Side-by-side comparison of temporal speckle contrast and confocal fluorescence

Supplementary Video Captions

Supplementary Note 1: Hardware-based compensation of coherence gate curvature (Model)

In our previous study[1], we used a fully computational approach to mitigate coherence gate curvature (CGC) artifacts which distort OCT/OCM image data. However, our approach relied on heuristics which only approximated the necessary distortion corrections in order to maintain computational efficiency. In this study, we employed a hybrid CGC mitigation method which physically corrected the majority of CGC via modified optical hardware and employed computational methods to correct for only small residual distortions. This section provides a model for our novel hardware-based mitigation method, while the next section provides results from a validation experiment.

Our microscope (depicted in Fig. S1) is a beam-scanning spectral domain OCM system. Similar to many research- and commercial-grade systems, our microscope uses a set of paired galvanometer mirrors to control scanning of the imaging beam across the lateral FOV. The use of paired galvanometers offers multiple benefits for OCM systems, including simple construction/alignment, compact design, and lower aberrations/losses (due to the reduction or elimination of relay optics ubiquitous to microscopes that use separate/distinct galvanometer mirrors). However, as is shown below, this simple and common 'paired galvanometer' convention in OCM system design can contribute substantial distortions (CGC) to 3D OCT/OCM images when using a wide lateral FOV (e.g., 1×1 mm^2 and greater). Thankfully, this fault of the paired galvanometer convention is easily compensated by our novel design, which incorporates a weak cylindrical lens into a 4F telescope relay. This cylindrical lens allows our system to maintain the benefits of a compact and simple paired galvanometer design, while simultaneously mitigating CGC artifacts.

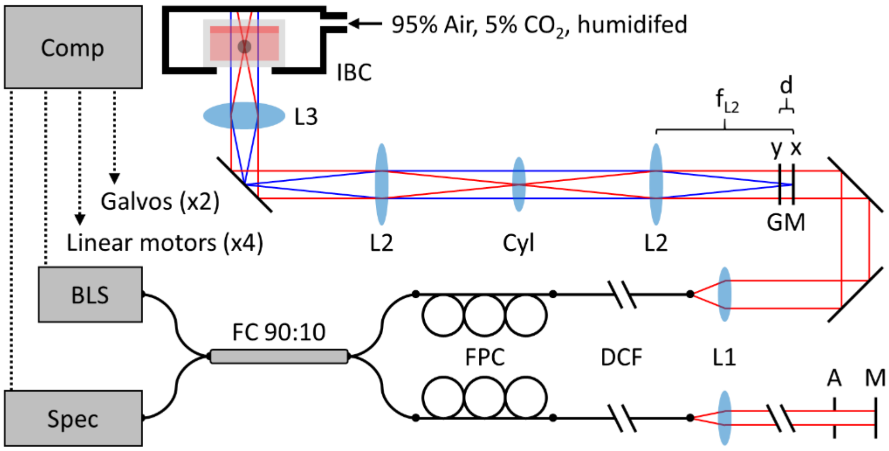

Figure S1. Diagram of our novel custom-built OCM imaging system for performing high-resolution, wide-FOV, low-distortion imaging (see the Methods in the main text for a detailed description of components). Comp: computer. BLS: broadband laser source. Spec: spectrometer + line scan camera. FC: fiber coupler (90% to reference arm, 10% to sample arm). FPC: fiber polarization controller. DCF: dispersion compensating fiber (each arm contains different lengths of fiber). A: aperture. M: mirror/retro-reflector. GM: galvanometer mirror (*x*,*y* denote the axis along which each mirror tilts). (Note: Only the *position* of the galvanometer mirrors *along the optical path* is depicted here.) d: galvanometer mirror separation (13.69 mm, imposed by housing). L1: Collimating lens (f_{L1}=19 mm). L2: Telescope lens (f_{L2}=100 mm). Cyl: Cylindrical lens ($f_x=\infty$, f_y=+700 mm, which helps to compensate for coherence gate curvature resulting from the physical separation between the *x* and *y* galvanometers along the optical axis). L3: Objective lens (idealized). IBC: incubating bio-chamber.

Here, we use ray transfer matrix analysis to investigate a simplified model of the sample arm of our OCM imaging system. Within the *xz*-plane, the 'chief' (i.e., central) ray of the collimated beam is parametrized by the position-angle vector $\langle r=0,\theta=0\rangle$. Upon reflection off of the *xz*-plane galvanometer mirror ('GM_x'), this ray is imparted with an angle of θ_x to become $\langle 0,\theta_x\rangle$. Propagating this angled ray through the sample arm up to a distance *z* after the objective lens ('Obj') results in the transformation:

$$\underbrace{\begin{bmatrix}1 & z\\0 & 1\end{bmatrix}}_{\text{Obj}\to z}\underbrace{\begin{bmatrix}1 & 0\\-1/f_o & 1\end{bmatrix}}_{\text{Obj}}\underbrace{\begin{bmatrix}1 & f+f_o\\0 & 1\end{bmatrix}}_{\text{TL2}\to\text{Obj}}\underbrace{\begin{bmatrix}1 & 0\\-1/f & 1\end{bmatrix}}_{\text{TL2}}\underbrace{\begin{bmatrix}1 & f\\0 & 1\end{bmatrix}}_{\text{Cyl}\to\text{TL2}}\underbrace{\begin{bmatrix}1 & 0\\-1/f_x & 1\end{bmatrix}}_{\text{Cyl}}\underbrace{\begin{bmatrix}1 & f\\0 & 1\end{bmatrix}}_{\text{TL1}\to\text{Cyl}}\underbrace{\begin{bmatrix}1 & 0\\-1/f & 1\end{bmatrix}}_{\text{TL1}}\underbrace{\begin{bmatrix}1 & f\\0 & 1\end{bmatrix}}_{\text{GM}_x\to\text{TL1}}\begin{bmatrix}0\\\theta_x\end{bmatrix} \tag{A.1a}$$

$$\begin{bmatrix}(1/f_o)(z-f_o) & -f_o+\left(f^2/f_o f_x\right)(z-f_o)\\(1/f_o) & \left(f^2/f_o f_x\right)\end{bmatrix}\begin{bmatrix}0\\\theta_x\end{bmatrix} \tag{A.1b}$$

$$\begin{bmatrix}(1/f_o)(z-f_o) & -f_o\\(1/f_o) & 0\end{bmatrix}\begin{bmatrix}0\\\theta_x\end{bmatrix},\ \text{for } f_x=\infty \tag{A.1c}$$

$$\begin{bmatrix}-f_o\theta_x\\0\end{bmatrix} \tag{A.1d}$$

where f denotes the focal length of the telescope lenses ('TL1' and 'TL2'), f_x denotes the focal length of the cylindrical lens ('Cyl') within the *xz*-plane, and f_o denotes the focal length of the (idealized) objective lens. Since GM_x is imaged to the back focal plane of the objective lens, the beam is scanned in an ideal telecentric fashion (i.e., the beam remains parallel to the optical axis while its lateral *x*-position is scanned proportionally to θ_x). Of course, deviations from this idealized model (e.g., thick lenses, aberrations in the telescope and/or objective, etc.) will cause non-ideal beam-scanning behavior at large scanning angles/lateral beam positions.

We further analyze the optical path length (OPL) traversed by the chief ray through the system. In the small-angle limit, propagation through air by a distance L (along the optical axis) at an angle θ results in an OPL accumulation of approximately $L\left(1+\theta^2/2\right)$. Likewise, transmission through a thin lens of focal length f at a lateral position r with respect to the optical axis accumulates an OPL (up to an additive constant) of approximately $-r^2/2f$. Following the path of the chief ray within the *xz*-plane, we derive a total accumulated OPL (up to an additive constant) of:

$$\begin{aligned}\text{OPL}_x(z,\theta_x) = & \underbrace{f\left(1+\theta_x^2/2\right)}_{\text{GM}_x\to\text{TL1}}-\underbrace{(f\theta_x)^2/2f}_{\text{TL1}}+\underbrace{f}_{\text{TL1}\to\text{Cyl}}-\underbrace{0}_{\text{Cyl},\,f_x=\infty}+\underbrace{f}_{\text{Cyl}\to\text{TL2}}-\underbrace{(f\theta_x)^2/2f}_{\text{TL2}}+\ldots\\ & \underbrace{(f+f_o)\left(1+(-\theta_x)^2/2\right)}_{\text{TL2}\to\text{Obj}}-\underbrace{(-f_o\theta_x)^2/2f_o}_{\text{Obj}}+\underbrace{z}_{\text{Obj}\to z}+\text{constant}\end{aligned} \tag{A.2a}$$

$$\text{OPL}_x(z,\theta_x)=4f+f_o+z+\text{constant} \tag{A.2b}$$

This corresponds to an ideal scenario, since OPL increases linearly with *z* and is not a function of scanning angle.

Within the *yz*-plane, we perform a similar analysis. The chief ray is imparted with an angle θ_y by the *yz*-plane galvanometer mirror ('GM_y') to become $\left\langle 0,\theta_y\right\rangle$. Propagation of this angled ray through the sample arm up to a distance *z* after the objective lens results in the transformation:

$$\underbrace{\begin{bmatrix}1 & z\\0 & 1\end{bmatrix}}_{\text{Obj}\to z}\underbrace{\begin{bmatrix}1 & 0\\-1/f_o & 1\end{bmatrix}}_{\text{Obj}}\underbrace{\begin{bmatrix}1 & f+f_o\\0 & 1\end{bmatrix}}_{\text{TL2}\to\text{Obj}}\underbrace{\begin{bmatrix}1 & 0\\-1/f & 1\end{bmatrix}}_{\text{TL2}}\underbrace{\begin{bmatrix}1 & f\\0 & 1\end{bmatrix}}_{\text{Cyl}\to\text{TL2}}\underbrace{\begin{bmatrix}1 & 0\\-1/f_y & 1\end{bmatrix}}_{\text{Cyl}}\underbrace{\begin{bmatrix}1 & f\\0 & 1\end{bmatrix}}_{\text{TL1}\to\text{Cyl}}\underbrace{\begin{bmatrix}1 & 0\\-1/f & 1\end{bmatrix}}_{\text{TL1}}\underbrace{\begin{bmatrix}1 & f-d\\0 & 1\end{bmatrix}}_{\text{GM}_y\to\text{TL1}}\begin{bmatrix}0\\\theta_y\end{bmatrix} \tag{A.3a}$$

$$\begin{bmatrix}(1/f_o)(z-f_o) & -f_o+\left(\left(f^2-f_y d\right)/f_o f_y\right)(z-f_o)\\(1/f_o) & \left(\left(f^2-f_y d\right)/f_o f_y\right)\end{bmatrix}\begin{bmatrix}0\\\theta_y\end{bmatrix} \tag{A.3b}$$

$$\begin{bmatrix}-f_o\theta_y+(z-f_o)\alpha\theta_y\\\alpha\theta_y\end{bmatrix},\ \text{for } \alpha=\left(\left(f^2-f_y d\right)/f_o f_y\right) \tag{A.3c}$$

where d denotes the separation between GM_x and GM_y along the optical axis. Here, since GM_y is *not* imaged to the back focal plane of the objective lens, the beam does *not* scan parallel to the optical axis (i.e., the output beam propagates at an angle which varies as a function of θ_y). Moreover, the lateral *y*-position of the beam varies as a function of both θ_y and depth *z*. These behaviors result in one of the hallmarks of CGC: a depth-dependent lateral magnification (i.e., non-telecentricity).

Likewise, following the path of the chief ray within the *yz*-plane, we derive a total accumulated OPL (up to an additive constant) of:

$$\begin{aligned}\mathrm{OPL}_y\left(z,\theta_y\right) = \underbrace{d}_{\mathrm{GM}_x\to\mathrm{GM}_y} + \underbrace{(f-d)\left(1+\theta_y{}^2/2\right)}_{\mathrm{GM}_y\to\mathrm{TL1}} - \underbrace{\left((f-d)\theta_y\right)^2/2f}_{\mathrm{TL1}} + \underbrace{f\left(1+\left(d\theta_y/f\right)^2/2\right)}_{\mathrm{TL1}\to\mathrm{Cyl}} - \underbrace{\left(f\theta_y\right)^2/2f_y}_{\mathrm{Cyl},\,f_y} + \ldots \\ \underbrace{f\left(1+\left(\left(d/f - f/f_y\right)\theta_y\right)^2/2\right)}_{\mathrm{Cyl}\to\mathrm{TL2}} - \underbrace{\left(\left(f+d-f^2/f_y\right)\theta_y\right)^2/2f}_{\mathrm{TL2}} + \underbrace{\left(f+f_o\right)\left(1+\left(-\theta_y\right)^2/2\right)}_{\mathrm{TL2}\to\mathrm{Obj}} + \ldots \\ -\underbrace{\left(\left(-f_o+d-f^2/f_y\right)\theta_y\right)^2/2f_o}_{\mathrm{Obj}} + \underbrace{z\left(1+\left(\left(-d/f_o+f^2/f_o f_y\right)\theta_y\right)^2/2\right)}_{\mathrm{Obj}\to z} + \text{constant}\end{aligned} \quad \text{(A.4a)}$$

$$\mathrm{OPL}_y\left(z,\theta_y\right) = \left[4f+f_o+z\right] + \left[-\alpha f_o + \left(z-f_o\right)\alpha^2\right]\theta_y{}^2/2 + \text{constant} \quad \text{(A.4b)}$$

where α is defined as in Eqn. (A.3c). Here, we observe that OPL varies quadratically as a function of θ_y. This results in the namesake 'curvature' of CGC. That is, flat surfaces will appear curved in the OCT image due to the fact that OCT microscopes use OPL measurements as a proxy for 'depth'.

Given this analysis, we hypothesized that CGC could be mitigated (within the confines of this idealized model) if $f_y = f^2/d$. This scenario would result in $\alpha = 0$, and would thus eliminate the quadratic terms in Eqn. (A.4b). In our system, the optimal focal length for our cylindrical lens was predicted to be $f_y=(100\text{ mm})^2/(13.69\text{ mm})\approx+730$ mm. However, since we used a non-optimal focal length of only +700 mm, our system was not expected to completely eliminate CGC. However, compared to the case of applying no correction (i.e., when $f_y=+\infty$), our system was *predicted* to reduce CGC (i.e., α) to approximately 5% of its original severity (i.e., when no compensation method is employed).

Supplementary Note 2: Hardware-based compensation of coherence gate curvature (Validation)

In order to assess the performance of our CGC-compensating system, we acquired images of a flat glass surface near the focal plane of our system, with and without the CGC-compensating cylindrical lens and spanning a 1×1 mm^2 lateral FOV. The resulting images (and apparent curvature of the glass surface) are depicted in Fig. S2. We then fit each surface (hereafter denoted by indices i=1 and i=2, respectively) with a quadratic polynomial:

$$z_i(x,y) = a_i x^2 + b_i xy + c_i y^2 + d_i x + e_i y + f_i \tag{A.5}$$

The terms a_i, b_i, and c_i record the apparent curvature of the glass surface and thus measure the severity of CGC. The terms d_i and e_i record the apparent tilt of the glass surface, which results from a combination of true sample tilt and/or non-ideal beam alignment within the sample arm optics (the latter of which can be compensated with an adjustable periscope placed prior to the galvanometers). The term f_i encodes the axial offset of the glass surface in the image.

The curvature terms for the imaged glass surfaces were found to have values (in units of μm^{-1}) of $a_1 = 4.585\times10^{-5}$, $b_1 = 0.015\times10^{-5}$, and $c_1 = -4.280\times10^{-5}$ for the uncorrected system, and $a_2 = -1.454\times10^{-6}$, $b_2 = 0.856\times10^{-6}$, and $c_2 = -2.415\times10^{-6}$ for the CGC-compensated system. In order to assess the total amount of CGC, we performed a rotation of the lateral coordinate system to make the cross-term b_i=0. Specifically, defining:

$$\begin{bmatrix} x \\ y \end{bmatrix} = \begin{bmatrix} \cos\theta_i & \sin\theta_i \\ -\sin\theta_i & \cos\theta_i \end{bmatrix} \begin{bmatrix} x' \\ y' \end{bmatrix} \tag{A.6}$$

we obtained the transformed curvature terms (A_i, B_i, and C_i) via:

$$\begin{bmatrix} A_i \\ B_i \\ C_i \end{bmatrix} = \begin{bmatrix} \cos^2\theta_i & -\cos\theta_i \sin\theta_i & \sin^2\theta_i \\ \sin 2\theta_i & \cos 2\theta_i & -\sin 2\theta_i \\ \sin^2\theta_i & \cos\theta_i \sin\theta_i & \cos^2\theta_i \end{bmatrix} \begin{bmatrix} a_i \\ b_i \\ c_i \end{bmatrix} \tag{A.7}$$

where $\theta_i = 0.5\arctan\left(b_i/(c_i - a_i)\right)$, yielding $A_1 = 4.585\times10^{-5}$, $B_1 = 0$, and $C_1 = -4.280\times10^{-5}$ for the uncorrected system, and $A_2 = -1.291\times10^{-6}$, $B_2 = 0$, and $C_2 = -2.578\times10^{-6}$ for the CGC-compensated system. The opposite signs of A_1 and C_1 correspond to positive CGC along one axis and negative CGC along the orthogonal axis, respectively. This corresponds to the scenario where the back focal plane of the objective lens is imaged by the 4F telescope to a plane that lies *between* the two galvanometer mirrors. In contrast, the matching negative signs of A_2 and C_2 indicate that the alignment of our CGC-compensated system was not optimal (i.e., the objective lens was placed too close to the telescope relay by a small margin). Despite this non-optimal alignment, our system managed to reduce CGC to *at most* $\max\left(|A_2|,|C_2|\right)/\min\left(|A_1|,|C_1|\right) \approx 6\%$ of its original severity, which is close to our theoretically predicted value of 5%. However, under optimal alignment conditions, we would expect to achieve the condition $A_i = -C_i$ for both systems (i.e., the back focal plane of the objective lens is imaged to a plane that lies at the mid-point between the galvanometer mirrors). Thus, defining the 'total' CGC of the system as $|C_i - A_i| = |2A_i| = |2C_i|$ under optimal alignment conditions, we estimate that our system is capable of reducing CGC to approximately $|C_2 - A_2|/|C_1 - A_1| \approx 1.5\%$ of its original severity. This exceeds our predicted improvement and could be attributable to spherical aberration of the telescope lenses causing their effective focal lengths to decrease at large scanning angles (and thus making them better-matched to the +700 mm focal length of the installed cylindrical lens).

Given these results, we conclude that our novel OCM imaging system provides both a simple and effective method to mitigate CGC artifacts which emerge from the use of paired galvanometer mirrors. As a result, our system substantially reduces a potential source of distortions which would otherwise corrupt deformation data that is essential to traction force microscopy. Since it remains difficult to realize an ideal system, computational methods should still be employed to mitigate small residual distortions. Such methods are detailed in the next section of this document.

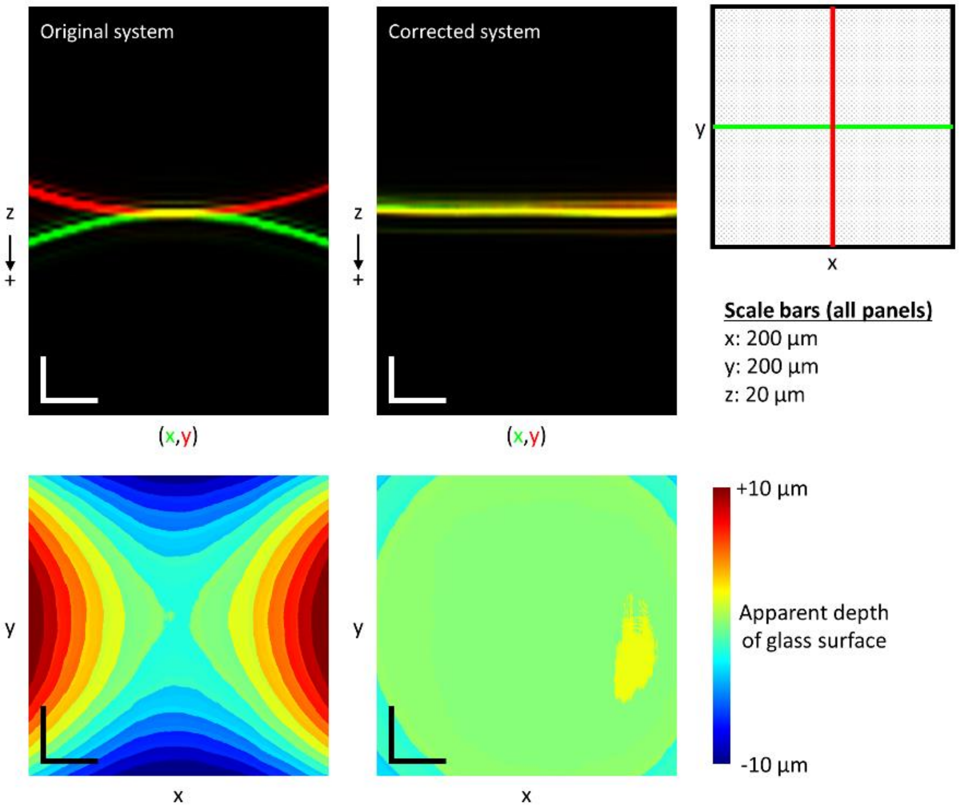

Figure S2. Visualization of coherence gate curvature (CGC) before (left) and after (right) hardware-based compensation. The top panels depict the OCM image of a (*physically* flat) glass-air interface in the *xz*- (green) and *yz*- (red) planes intersecting the origin of the lateral FOV. Any *observed* curvature of the surface in these panels is a consequence of CGC distortions. The bottom panels depict the observed apparent depth (i.e., the optical path length) of the surface as a function of lateral position. In the original system, CGC causes image distortions which result in the (physically flat) glass surface appearing as a hyperbolic paraboloid surface. In the corrected system, the glass surface appears nearly flat (with a residual weak paraboloid shape due to non-optimal alignment). See text for details.

Supplementary Note 3: OCT image reconstruction procedure

Here, we provide detailed flow charts and equations for our OCT image reconstruction procedure. Note that more detailed explanations/justifications underlying many of the operations below may be found in Supp. Ref. [1].

Depth-selective OCT volume reconstruction

OCT images were reconstructed from spectral data by applying the standard operations of background subtraction, spectrum resampling, dispersion compensation, and the Fourier transform. In order to enable the reconstruction of small/specific depth ranges (as opposed to the full depth range of the available axial FOV), a matrix multiplication-based method analogous to previously reported algorithms[2-4] was employed. Specifically, for a (post-background subtraction) A-scan of spectral data $v(k_b, x, y)$ for $b \in \{1, 2, \ldots, 2048\}$, the OCT image signal $s(z_a, x, y)$ at a given integer depth index a was computed via:

$$s(z_a, x, y) = \sum_{b=1}^{2048} v(k_b, x, y) \exp\left(-j\left(2k_b z_a + \alpha_2\left(k_b - \bar{k}\right)^2 + \alpha_3\left(k_b - \bar{k}\right)^3\right)\right) = \sum_{b=1}^{2048} \Theta_{ab} v(k_b, x, y) \quad \text{(B.1)}$$

where a is constrained to the set of integers $a \in \{1, 2, \ldots, 2048\}$, $j = \sqrt{-1}$, k_b denotes the wavenumber of the bth spectrometer pixel (determined by manufacturer-generated data sheets), $\bar{k} = (k_1 + k_{2048})/2$, $z_a = \Delta z(a - 1025)$ for $\Delta z = (2047/2048)(\pi / |k_{2048} - k_1|)$, and (α_2, α_3) denote (manually calibrated) dispersion compensation parameters. Note that Δz corresponds to the height of each voxel in terms of optical path length (OPL). The physical height of a given voxel is $\Delta z / n$, where n is the local refractive index of the imaged medium.

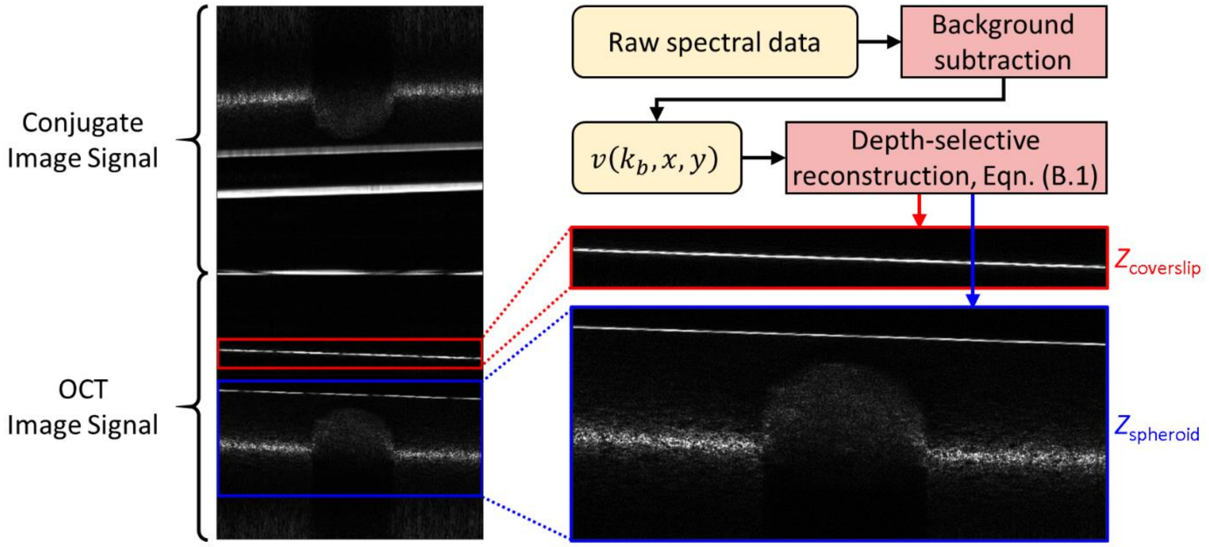

Figure S3. Depth-selective OCT image reconstruction and regions of interest. See text for details.

There were two regions of interest in the imaged volumes. The first is a narrow range of depths $z_a \in Z_{\text{coverslip}}$ which contain the coverslip surface (i.e., the base of the sample dish) across all time-points. The second is a larger range of depths $z_a \in Z_{\text{spheroid}}$ which contain the spheroid and surrounding regions across all time-points. Examples of these regions are depicted in Fig. S3.

Coherence gate curvature removal and phase registration

As discussed in the previous sections of this supplementary document, coherence gate curvature (CGC) is a consequence of non-idealities in imaging system design/implementation and/or sample positioning which distort OCT images. A flat surface may appear to be tilted and/or curved in OCT images acquired by a system corrupted by CGC. Unlike our previously reported methods[1], which used a single computational procedure for mitigating CGC, here we employed a three-pronged hybrid approach to obtain more robust results. The majority of CGC was removed via the cylindrical lens incorporated into our novel OCM imaging system (discussed previously, and depicted in Fig. S1). Residual CGC was removed computationally in two steps, hereafter called 'coarse' and 'fine' CGC removal. The latter step simultaneously performs phase registration (another key operation for OCT image reconstruction). CGC calibration and removal were performed independently for each image and time-point (except where otherwise noted).

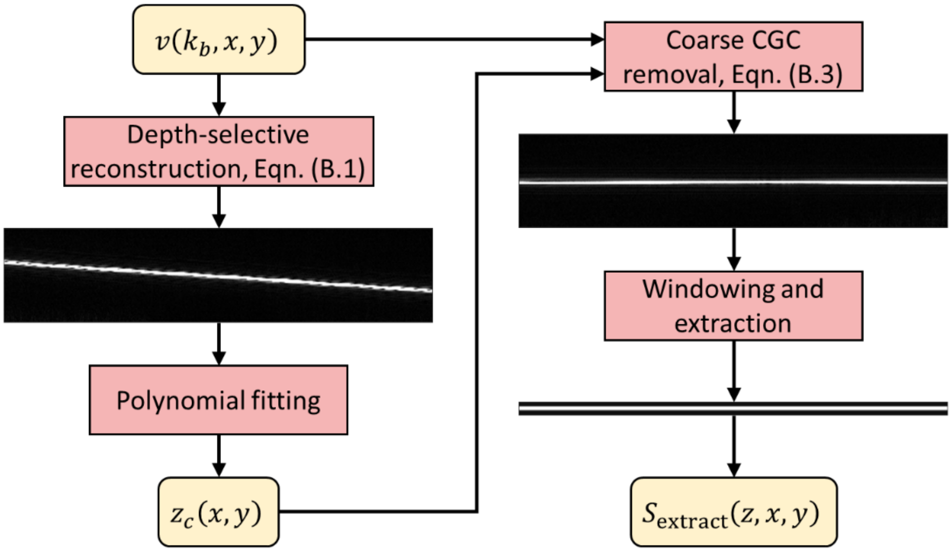

Figure S4. Calibration procedure for both 'coarse' (left column) and 'fine' (right column) coherence gate curvature removal (and phase registration). See text for details.

The first step is a calibration stage (depicted in Fig. S4), which performs calibrations for both coarse and fine CGC removal (and phase registration). An OCT image of the $Z_{\text{coverslip}}$ region is reconstructed according to Eqn. (B.1). The laterally varying axial position of the coverslip surface (in terms of OPL) is then approximated by the function:

$$z_c(x, y) = c_{xx}x^2 + c_{xy}xy + c_{yy}y^2 + c_x x + c_y y + c_0 \tag{B.2}$$

This completes the calibration stage for coarse CGC removal. Next, the $Z_{\text{coverslip}}$ region is reconstructed *again* while applying coarse CGC removal via:

$$\hat{S}(z_a, x, y) = \sum_{b=1}^{2048} \Theta_{ab} v(k_b, x, y) \exp\left(-j2k_b\left(z_c(x, y) - z_0\right)\right) \tag{B.3}$$

where z_0 is defined as the value of c_0 obtained for the *first* time-point in the time-lapse data set. In the resulting 3D image, the coverslip will appear *nearly* flat and level, and will be centered at the OPL position $z = z_0$. Note that by using the same value of z_0 for all time-points, all reconstructed images in a given time-series will be automatically registered/aligned along the *z*-axis. The coarsely-corrected image is next cropped along the *z*-axis such that only ~11-21 voxels remain along the *z*-axis centered around the position $z = z_0$. This cropped volume will be referred to as $S_{\text{extract}}(z, x, y)$. This completes the calibration stage for fine CGC removal and phase registration.

The cropped volume $S_{\text{extract}}(z,x,y)$ is used to perform both fine CGC removal and phase registration. Note that although this volume was derived from the $Z_{\text{coverslip}}$ region of the sample, it may be applied to the reconstruction of any sub-region from the full OCT data set (e.g., the Z_{spheroid} region). In order to perform fine CGC removal and phase registration to a volume $P(z,x,y)$, $S_{\text{extract}}(z,x,y)$ is first zero-padded along the *z*-axis until it is of the same size as $P(z,x,y)$. Then, the following operation is performed:

$$P_r(z,x,y) = FT^{-1}_{z\to q_z}\left[FT_{z\to q_z}\left[P(z,x,y)\right]\exp\left(-j\arg\left(FT_{z\to q_z}\left[S_{\text{extract}}(z,x,y)\right]\right)\right)\right] \tag{B.4}$$

where $FT_{z\to q_z}$ and $FT^{-1}_{z\to q_z}$ denote the forward and inverse Fourier transform along *z*, respectively, and $P_r(z,x,y)$ denotes the output volume (which lacks CGC and is phase-registered). This procedure is depicted in Fig. S5.

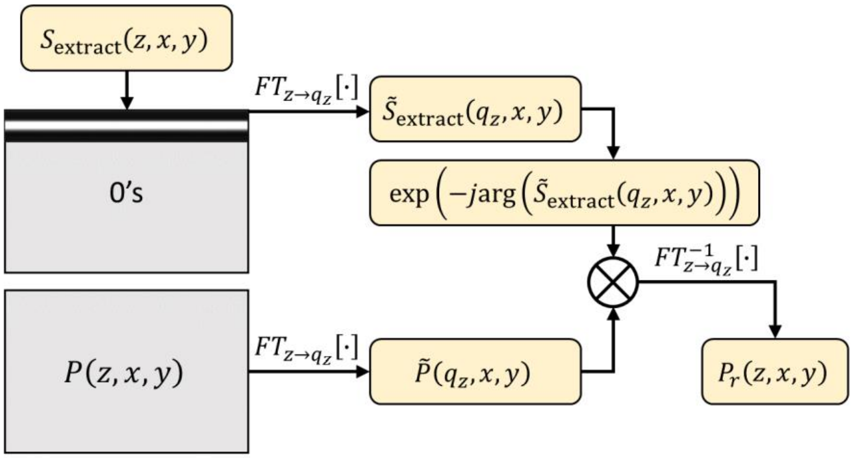

Figure S5. Fine coherence gate curvature removal and phase registration. See text for details.

Focal plane curvature removal

Focal plane curvature (FPC) is another consequence of non-optimal system design and/or sample positioning. This results in the focal 'plane' appearing tilted and/or curved, even after CGC removal has been performed (i.e., the curvature of the focal plane does not necessarily match that of the 'coherence gate'). This results in OCT images which exhibit a laterally-varying point spread function (PSF), which conventional refocusing algorithms (such as the computational adaptive optics (CAO) procedure used in this study) do not accommodate, due to the assumption of a laterally-invariant PSF. Alternative formulations of CAO which account for lateral variation do exist[5,6]. However, such procedures can be computationally expensive and complicated to calibrate and perform. In our previous study[1], we demonstrated an alternative procedure for FPC mitigation which enables the use of computationally efficient CAO algorithms that leverage lateral invariance assumptions.

Unlike our previous study[1] (which measured/calibrated FPC from images of the sample directly), here we used a separate calibration data set (which is described in the Methods of the main text). This calibration data set was used to obtain a measurement of FPC, which was subsequently applied to all images in the corresponding time-series. The reason for this change in procedure is a consequence of the new experimental settings encountered in this study. In our previous study, we imaged isolated cells which occupied a miniscule fraction of the total volumetric FOV. Therefore, FPC could be readily measured from images of the sample. However, the spheroids imaged in this study occupied a large fraction of the volumetric FOV, and substantially obstructed the focal plane across much of the FOV. This prevented automated measurements of FPC using only images of the spheroid(s). The calibration data set used in this study contained no spheroid (but was instead merely acquired *near* the spheroid). This provided a clear and unobstructed view of the geometry of the focal 'plane' across the entire FOV. The FPC measured from this data set was assumed to be a suitable proxy for FPC calibration of the spheroid images in a given time-series.

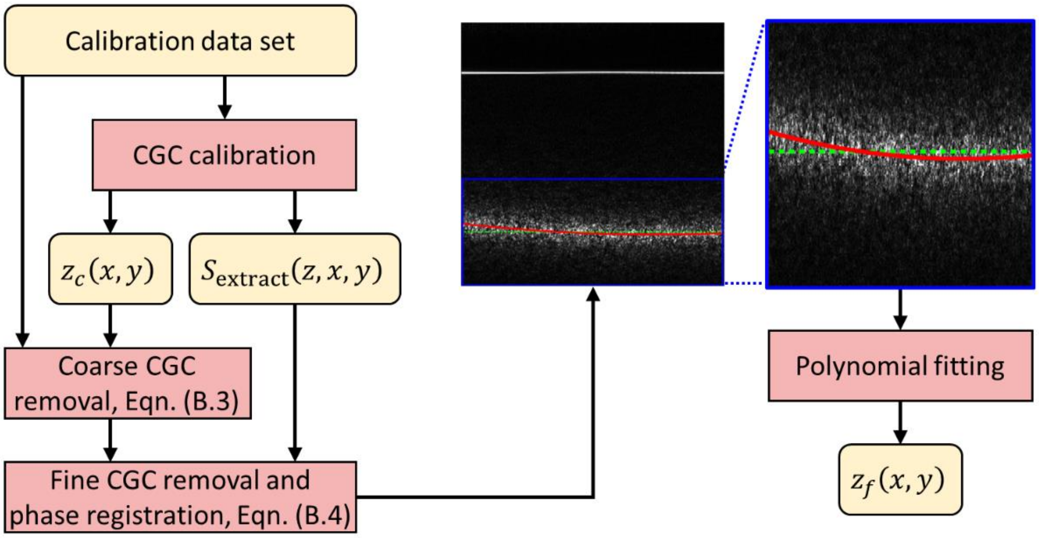

Figure S6. Calibration procedure for focal plane curvature removal. Note that the focal plane appears curved even though the coverslip surface appears flat and level. See text for details.

FPC calibration was performed as depicted in Fig. S6. First, CGC calibration is performed on the calibration data set. Then, the 'focal plane region' of the sample (which is analogous to the $Z_{spheroid}$ region, but with no spheroid present, given the nature of the calibration data set) is reconstructed while applying coarse CGC removal, fine CGC removal, and phase registration. The laterally varying position of the focal 'plane' (in terms of OPL) is approximated via:

$$z_f(x,y) = f_{xx}x^2 + f_{xy}xy + f_{yy}y^2 + f_x x + f_y y + f_0 \tag{B.5}$$

This completes the calibration stage for FPC removal. In order to apply FPC removal (as depicted in Fig. S7), the $Z_{spheroid}$ region of the spheroid images are first reconstructed via:

$$\hat{S}(z_a,x,y) = \sum_{b=1}^{2048} \Theta_{ab} v(k_b,x,y) \exp\left(-j2k_b\left(z_c(x,y)-z_0\right)\right) \exp\left(-j2k_b\left(z_f(x,y)-\bar{z}_f\right)\right) \tag{B.6}$$

where $\bar{z}_f$ is defined as the mean value of $z_f(x,y)$ across the lateral FOV. Note that this formula is nearly identical to that for coarse CGC removal (Eqn. (B.3)), with an extra phase term added for performing FPC removal as well. Following this reconstruction, fine CGC removal and phase registration are performed via Eqn. (B.4) to obtain a phase registered image in which the focal plane appears flat and level (although the coverslip surface appears curved, due to the mismatch between CGC and FPC). This volume, which we will call '$S_r(z,x,y)$', is compatible with digital refocusing procedures which assume a laterally invariant PSF[1,7].

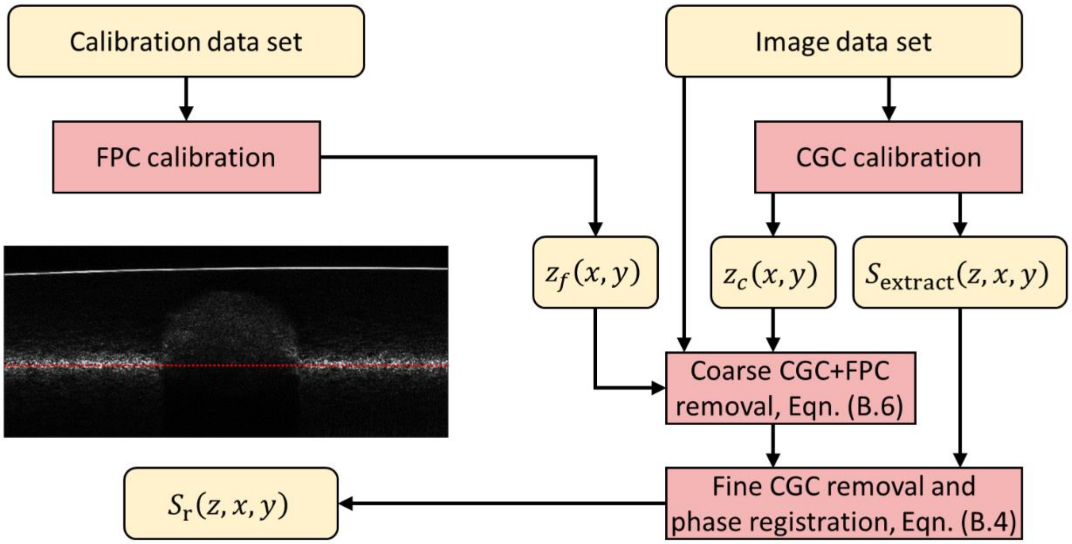

Figure S7. Procedure for joint application of coarse CGC removal, fine CGC removal, phase registration, and FPC removal. Note that this results in an image where the focal plane is flat and level, but the coverslip surface is not. See text for details.

Bulk demodulation

As discussed in our previous study[1], optical misalignments and/or sample tilt can result in the OCT image signal undergoing a bulk phase modulation across the lateral dimensions. Failing to account for this modulation can result in depth-dependent shearing artifacts which emerge after applying CAO. Calibration of the bulk modulation in the system was performed as depicted in Fig. S8. First the 'full FOV image' (defined in the Methods section of the main text) from the first time-point in a given time-series is reconstructed using the FPC removal procedure detailed previously. Next, any depths containing a glass surface are windowed out from the volume or set to zero (since the signal from such reflective surfaces can overwhelm/corrupt the calibration procedure). The remaining volume then undergoes the following operation:

$$M(q_x, q_y) = \int \left| FT_{3D}\left[S_r(z,x,y)\right]\right| dq_z \tag{B.7}$$

where FD_{3D} denotes the 3D forward Fourier transform operation. The resulting real-valued function $M(q_x,q_y)$, which is depicted in the upper-right panel of Fig. S8, approximates the lateral spatial frequency content of the image signal. Given the use of Gaussian beams in our system, $M(q_x,q_y)$ has an approximately Gaussian profile and is off-centered with respect to the origin (q_x=0,q_y=0) due to the bulk phase modulation of the image signal (which we wish to measure and remove). Performing peak-finding along the q_x- and q_y-axes results in a measurement of the bulk modulation coefficients: ($q_{x,0}$,$q_{y,0}$).

Once this calibration was performed, all volumes in the time-lapse data set were demodulated according to:

$$S_d(z,x,y) = S_r(z,x,y)\exp\left(-j\left(q_{x,0}x + q_{y,0}y\right)\right) \tag{B.8}$$

In doing so, it was assumed that the bulk modulation was constant across the entire time-lapse data set.

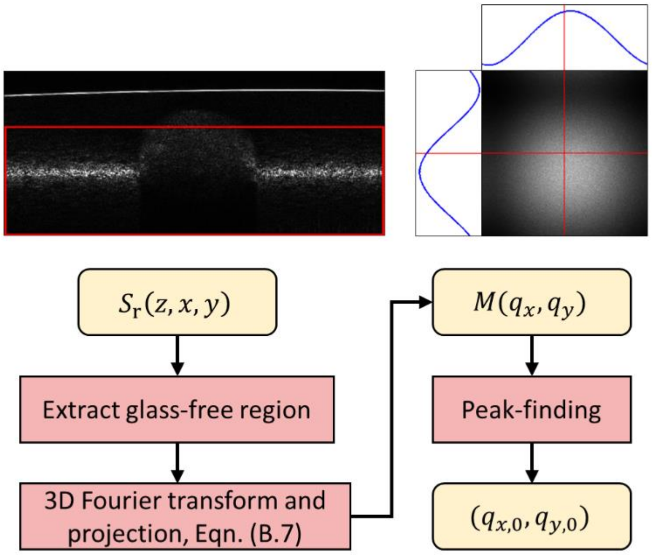

Figure S8. Calibration procedure for bulk demodulation. See text for details. The red boxed region in the upper-left panel shows the region that is used to perform the calibration (note that it excludes the glass surface which appears near the top of the image). $M(q_x,q_y)$ is shown in the upper right panel. The intersection of the two red lines denotes the origin of the lateral spatial frequency domain.

Computational adaptive optics

Computational adaptive optics[7] (CAO) is used to compensate for depth-dependent degradation of the lateral resolution of the OCT system. Here, we assumed that this degradation was due entirely to defocus (and not higher-order optical aberrations). In order to perform CAO, first the depth of the focal plane must be calibrated. For a given time-point, the 'full FOV image' was reconstructed using the procedures described above (CGC removal, FPC removal, phase registration, and bulk demodulation). Next the average axial intensity profile was computed from the 3D region of interest depicted in Fig. S9. (Note that this region excludes both the spheroid body as well as any glass surfaces which appear in the image.) Curve-fitting was performed in order to find the peak of this axial intensity profile and thus obtain the depth z_{focus} of the focal plane (in terms of physical distance). This position was used for both the full FOV and reduced FOV images of the spheroid (defined in the Methods section of the main text) for the given time-point.

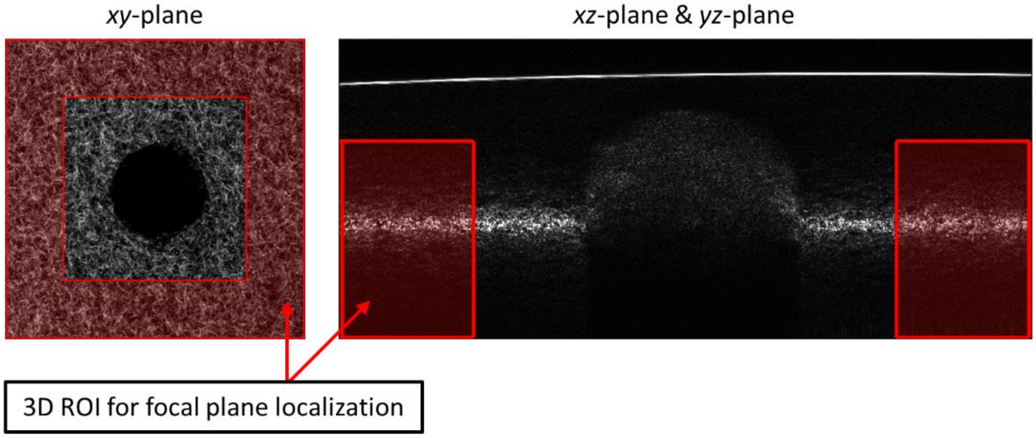

Figure S9. 3D region of interest for axial localization of the focal plane. The image shown in the right-hand panel intersects the origin of the lateral FOV. See text for details.

Next, defocus-compensation was performed via:

$$S_f(z,x,y) = FT^{-1}_{(x,y)\to(q_x,q_y)}\left[FT_{(x,y)\to(q_x,q_y)}\left[S_d(z,x,y)\right]\exp\left(-j\left(z-z_{\text{focus}}\right)\sqrt{\left(2n\bar{k}\right)^2-q_x^{\,2}-q_y^{\,2}}\right)\right] \quad \text{(B.9)}$$

where $FT_{(x,y)\to(q_x,q_y)}$ and $FT^{-1}_{(x,y)\to(q_x,q_y)}$ denote the forward and inverse 2D Fourier transform across the lateral dimensions, respectively, n denotes the refractive index of the sample (here, n=1.34 was used), and $\bar{k}$ is defined as before (and corresponds to the central wavenumber measured by the spectrometer of imaging system). Following this operation, the magnitude of the (complex-valued) image signal was normalized with respect to z. (Note that the normalized image remains complex-valued!) The results of this procedure are depicted in Fig. S10.

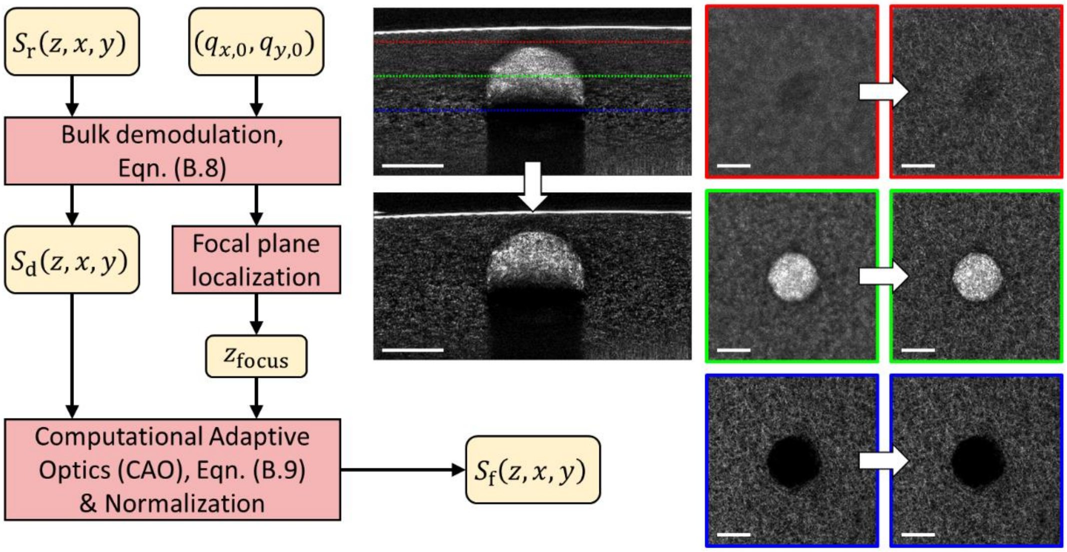

Figure S10. Procedure for defocus compensation with computational adaptive optics. See text for details. Panels at the right depict *en face* planes before and after CAO at the depths indicated by the three colored lines spanning the top-middle panel. Red/top: 225 µm above the focal plane. Green/middle: 112.5 µm above the focal plane. Blue/bottom: Focal plane. Scale bars = 200 µm.

Focal plane curvature restoration

In this study, we applied the same 'ideal coordinate system' heuristic established in our previous study[1]. That is, the ideal/final coordinate system for our reconstructed OCT images was assumed/defined as the one in which the coverslip surface appears both flat and level. However, CAO could only be performed while the *focal plane* was flat and level. Therefore, the final step in OCT image reconstruction is to *restore* the focal plane curvature to its original state, which returns the coverslip surface to its 'ideal' flat and level state.

For a reconstructed OCT volume with $N \le 2048$ pixels along the axial dimension (recall that our spectrometer camera had 2048 pixels), a frequency-axis vector $\mathbf{q}_z \in \mathbb{R}^{N\times 1}$ is defined such that:

$$q_{z,i} = \left(\frac{2\pi}{|\Delta z|}\right)\left(\frac{i-1}{N}\right), \text{ for } i \in \{1,2,...,N\} \quad \text{(B.10)}$$

where Δz is defined (in terms of OPL) as before. Then, FPC restoration is performed via:

$$FT^{-1}_{z\to q_z}\left[FT_{z\to q_z}\left[S_f(z,x,y)\right]\exp\left(-jq_z\left(z_f(x,y)-\bar{z}_f\right)\right)\right] \quad \text{(B.11)}$$

The final reconstructed image that results from this procedure is depicted in Fig. S11. Note that this volumetric image signal is a complex-valued function. All subsequent image processing (detailed in the Methods section of the main text) were performed on the *magnitude* of this function.

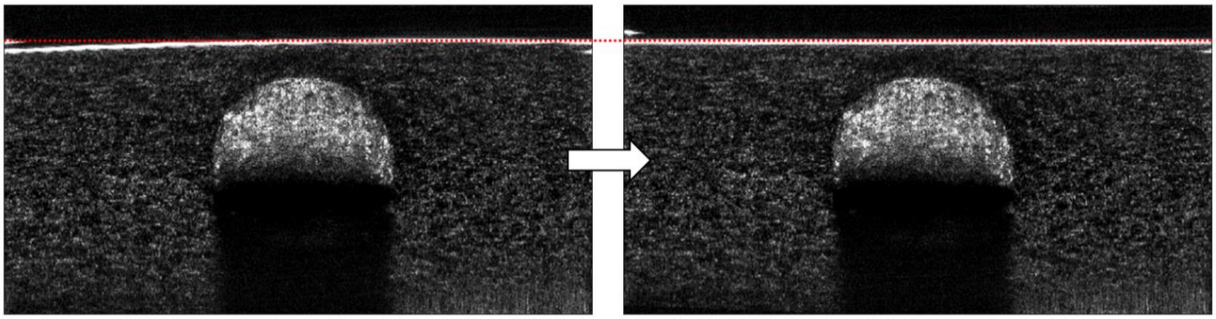

Figure S11. Focal plane curvature restoration. The CAO-processed and intensity-normalized image (left) is transformed to a final output image (right) wherein the coverslip surface appears both flat and level. See text for details.

Supplementary Note 4: Displacement tracking noise floor

In order to estimate the noise floor of our displacement tracking procedure, we performed a 'self-mapping' test. Our 'burst' imaging protocol (described in the Methods) yielded several redundant images (the 8 'reduced FOV' volumes) of the collagen substrate at each time-point. Due to the short duration over which these images were acquired (less than 2 minutes for a given 'burst'), the observed collagen structure is expected to undergo only negligible deformations. Therefore, if displacement tracking is performed between images obtained from a single time-point, the output displacement field estimate will be dominated by noise artifacts emerging from a combination of imaging system noise and limitations of the tracking algorithm.

To this end, two modified 'mean projections' were obtained (analogous to Eqn. (1) in the Methods) at time *t*=0:

$$\bar{S}_1(x,y,z,t=0)=\left(\frac{1}{4}\right)\sum_{i=1}^{4}\left|S_{2i-1}\left(x,y,z,t=0\right)\right| \quad \text{(C.1)}$$

$$\bar{S}_2(x,y,z,t=0)=\left(\frac{1}{4}\right)\sum_{i=1}^{4}\left|S_{2i}\left(x,y,z,t=0\right)\right| \quad \text{(C.2)}$$

That is, the two 'mean projections' were obtained from non-overlapping subsets of the 8 'reduced FOV' volumes $\left\{S_1,...,S_8\right\}$ acquired for the given time-point. (It should be noted that the imaging noise present in both $\bar{S}_1$ and $\bar{S}_2$ is expected to be higher than that of $\bar{S}$ from Eqn. (1) due to each signal being generated from only half the total images used to generate $\bar{S}$. Therefore, the displacement tracking noise floor estimates provided here may be larger than the true displacement tracking noise floor due to larger imaging noise contributions.) The volumes $\bar{S}_1$ and $\bar{S}_2$ were then converted into 'collagen' channel images via the procedure defined in the Methods (i.e., the volumes were subjected to down-sampling, and voxels corresponding to 'cells' were set to 0). Displacement tracking was then performed via the *imregdemons* function (using the same parameters defined in the Methods) to estimate the displacement field:

$$\mathbf{u}(x,y,z)=\left\langle u_x(x,y,z),u_y(x,y,z),u_z(x,y,z)\right\rangle \quad \text{(C.3)}$$

which maps $\bar{S}_1$ to $\bar{S}_2$. The displacement tracking noise floor for each dimension (*x*, *y*, and *z*) was estimated by computing the standard deviation of the associated displacement field components (u_x, u_y, and u_z, respectively) throughout the collagen substrate within a depth range spanning the 'surface' of the spheroid (i.e., the edge closest to the coverslip bottom of the petri dish) to the 'equator' (middle) of the spheroid. These noise floors, $(\sigma_x,\sigma_y,\sigma_z)$ were computed for 6 ASC monoculture spheroids, and 6 ASC + MCF10AT1 co-culture spheroids. The average noise floors computed across these samples were (38 nm, 35 nm, 30 nm) for the monoculture spheroids and (43 nm, 42 nm, 39 nm) for the co-culture spheroids. The average noise floor σ (reported in the Methods) was defined as $\sigma=(\sigma_x+\sigma_y+\sigma_z)/3$, yielding σ = 34 nm and σ = 41 nm for the monoculture and co-culture cases, respectively. Normalizing σ by the (down-sampled, isotropic) voxel side-length of the images used for displacement tracking (1.88 μm for monoculture spheroids and 2.34 μm for co-culture spheroids, as described in the Methods), yields a displacement tracking noise floor of approximately 0.02 voxels for both cases.

Supplementary Note 5: Quantification of displacement tracking performance at varying spatial scales

In order to measure the ability of the *imregdemons* function to track deformations in our collagen substrates, we performed a virtual displacement recovery experiment. In brief, volumetric images of collagen were virtually/numerically deformed with a series of known displacement fields. The *imregdemons* function was then applied to estimate the displacement field that would map/register the undeformed images to the deformed images. The estimated displacement fields were then used to quantify how well the *imregdemons* function recovered the true displacement fields across a range of spatial scales.

Before detailing our methods and results, we would like to note that one obvious alternative to our virtual experiment would be to image collagen substrates containing embedded scattering beads, followed by a comparison of the displacement fields estimated via *imregdemons* versus those generated by a more conventional method such as particle tracking (e.g., as performed in Supp. Ref. [1]). However, this type of experiment could pose multiple challenges.

First, in order for the embedded beads to be detectable within the background medium, the beads would need to exhibit strong scattering signals in comparison to the collagen fibers. Since Thirion's Demons algorithm leverages image gradients to perform tracking[8,9], the behavior of *imregdemons* could be altered in the presence of these strong scattering signals distributed throughout the volumetric FOV. Note that setting the intensities of voxels containing beads to zero or another constant value could still pose a bias problem, due to the presence of strong image *gradients* at the *boundaries* of bead-containing regions. (We note that in the experiments of the main manuscript, a similar problem may alter tracking performance near the spheroid-collagen interface, since pixels containing cells were set to 0 in the 'collagen channel' images. However, these effects would be expected to be isolated near the spheroid surface, whereas the effects of 'bead-collagen interfaces' would be distributed *throughout* the entire volume of the collagen.) As a consequence, the resulting displacement field estimates may not be representative of those that would be generated in the presence of collagen without embedded beads.

Second, in order to assess algorithm performance at a given spatial scale, the average bead spacing would need to be on the order of (or smaller than) the desired spatial scale. Probing algorithm performance at small spatial scales would therefore require the embedded beads to be very dense, which could further exacerbate the potential for the algorithm to track primarily beads, rather than collagen fibers.

Our virtual experiment (detailed below) allows us to avoid these issues and assess the tracking of collagen fibers without the influence of intervening bead signals. The use of virtual displacements has the added benefit of allowing us to probe algorithm performance at specific spatial scales and/or amplitudes with known values, rather than contending with the uncertain multi-scale/multi-amplitude displacement fields that would be generated by cells in a physical experiment.

To perform our displacement recovery experiment, first, a collagen substrate (with no cells in the FOV) was imaged using the 'burst protocol' outlined in the Methods of the main manuscript. The 8 'reduced FOV' volumes were used to generate 2 distinct volumetric images, $\bar{S}_1$ and $\bar{S}_2$, as defined by Eqns. (C.1) and (C.2) in Supplementary Note 4. As a result, we obtained 2 nearly identical images of the collagen substrate with distinct noise patterns representative of the actual noise encountered in the primary experiments of our manuscript. The volumes $\bar{S}_1$ and $\bar{S}_2$ were then converted into 'collagen' channel images via the procedure defined in the Methods (i.e., the volumes were subjected to down-sampling; no voxels were set to 0, since no cells were present in the FOV for these images). $\bar{S}_1$ and $\bar{S}_2$ were then truncated to a final size of 750×750 μm^2 (400×400 voxels) along the lateral dimensions, and 470 μm (250 voxels) along the axial dimension (centered on the focal plane). (This step helped to limit the volume size so that our displacement tracking could be completed for all test cases within a reasonable time frame.)

The truncated volume, $\bar{S}_1$ served as the 'undeformed' or 'reference' image in all tests. The truncated volume $\bar{S}_2$ served as the 'deformed' or 'test' image, and was subjected to various artificial deformations in each test. Specifically, $\bar{S}_2$ was subjected to uniaxial displacement fields along the *x*-, *y*-, or *z*-dimension. (We note that only uniaxial displacements/strains were tested here, since probing multi-dimensional displacements/strains across the full range of

spatial scales that we explored would require computation times that are infeasible for the current study.) For example, for uniaxial deformation applied along the *x*-dimension, the deformed volume was given by:

$$\bar{S}_{2,deformed}(x,y,z) = \bar{S}_2(x + u_{x,n}(x), y, z), \tag{D.1}$$

where the above equation was evaluated via the MATLAB function *interpn* using the 'spline' method. The displacement function $u_{x,n}(x)$ (or analogously, $u_{y,n}(y)$ or $u_{z,n}(z)$ for tests along their respective dimensions) was defined to be a sinusoid of the form:

$$u_{x,n}(x) = \frac{A}{nf_{0,x}} \sin\left(nf_{0,x}x\right), \tag{D.2}$$

where A denotes the amplitude of the *strain* induced by the displacement field, $f_{0,x} = 2\pi/X$ for X the length of the volumetric image along the *x*-dimension, and n denotes the number of spatial periods (cycles) in the sinusoidal 'displacement wave' spanning the FOV. Here, A was chosen to be 0.05 (that is, all applied displacement fields were designed to induce a 'strain wave' with an amplitude of 5% strain). Individual tests of the *imregdemons* function were performed along each dimension at various values of n (specifically, for $n \in \{1, 2, \ldots, 40\}$). It is important to note that we chose to use a constant strain amplitude (as opposed to a constant displacement amplitude) since strain is the 'true' measure of material deformation. (For example, a material subjected to a rigid translation experiences displacement, but no internal deformation/strain.) Moreover, using a constant displacement amplitude could yield non-physical deformations at small spatial scales (i.e., at large values of n). Examples of numerically deformed test images are depicted in Figure S12 for different values of n. The two most important features to note are that 1) the displacement field amplitude decreases as *n* increases (in order to maintain a 5% strain amplitude), and 2) each spatial period of the 'displacement wave' contains 2 strain regimes, a compression region and an expansion region, respectively.

Using the same parameters defined in the Methods (pyramid levels = 3, iterations per pyramid level = 100, and accumulated field smoothing = 2), the *imregdemons* function was applied to obtain an estimated displacement field $\hat{u}_{x,n}(x,y,z)$ (or analogously, $\hat{u}_{y,n}(x,y,z)$ or $\hat{u}_{z,n}(x,y,z)$ for the tests along the *y*- and *z*-dimensions, respectively) that maps the reference volume $\bar{S}_1$ to the test volume $\bar{S}_{2,deformed}$. The amplitude of the 'recovered' displacement field was then obtained via:

$$U_{rec,x,n} = \frac{2}{X}\int_0^X \left(\frac{1}{YZ}\int_0^Z \int_0^Y \hat{u}_{x,n}(x,y,z)\,\mathrm{d}y\,\mathrm{d}z\right) \sin\left(nf_{0,x}x\right)\,\mathrm{d}x, \tag{D.3}$$

where (X,Y,Z) are the length of the volumetric image along the *x*-, *y*-, and *z*-dimensions, respectively. Analogous formulas were used to perform amplitude recovery for the *y*- and *z*-deformation tests. The above operation is equivalent to 1) computing the average recovered uniaxial displacement field, followed by 2) computing the Fourier series coefficient at the spatial period of the true applied displacement field. The root-mean-squared-error (RMSE) was likewise computed via:

$$RMSE_{x,n} = \sqrt{\frac{1}{XYZ}\int_0^Z \int_0^Y \int_0^X \left(\hat{u}_{x,n}(x,y,z) - u_{x,n}(x)\right)^2 \,\mathrm{d}x\,\mathrm{d}y\,\mathrm{d}z} \tag{D.4}$$

Again, analogous formulas were used to compute $RMSE_{y,n}$ and $RMSE_{z,n}$ for their respective tests. Taking the RMSE to be the 'noise floor' of the estimated displacement field, we observed that the RMSE for each dimension $(\sigma_x, \sigma_y, \sigma_z)$ took a mean value of (70 nm, 67 nm, 69 nm), or approximately 0.04 voxels, and a maximum value of (92 nm, 89 nm, 85 nm), or approximately 0.05 voxels. These values are a factor of 2-3 times larger than those reported from the test in Supplementary Note 4, which suggests that measuring the noise floor via a 'self-mapping' (zero-displacement) test alone may be insufficient to fully characterize the behavior of the tracking algorithm.

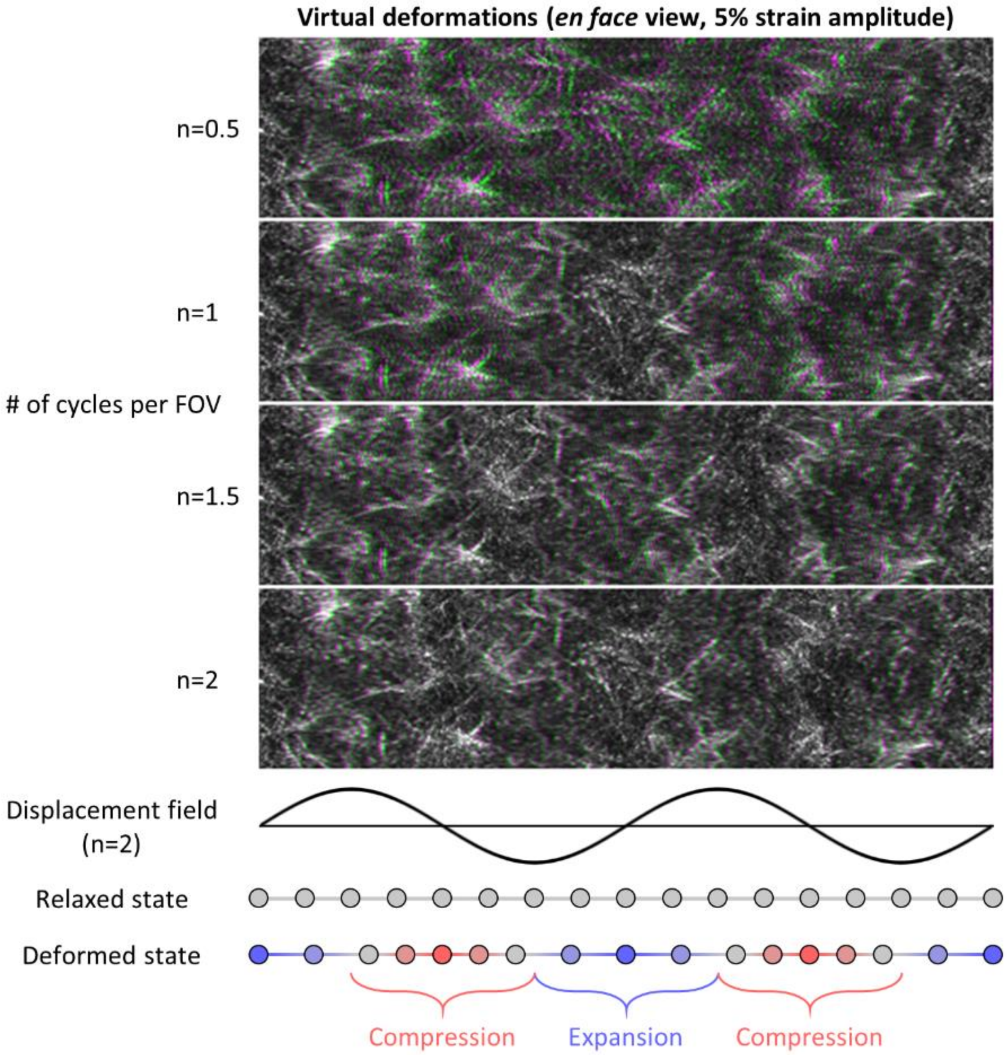

Figure S12. Examples of numerically deformed test images. The four panels above depict the same 750×188 μm^2 region of the sample. The purple channel shows the undeformed reference image, whereas the green channel shows the numerically deformed test image, which has been subjected to a horizontally-aligned sinusoidal displacement field containing n = 0.5, 1, 1.5, and 2 spatial periods (cycles) across the FOV, respectively. Due to our constant (5%) strain amplitude convention, the amplitude of the displacement field decreases as n increases. Each spatial period of the sinusoidal 'displacement wave' contains 2 distinct strain regimes, as illustrated at the bottom for n=2.

Recovered displacement amplitudes and RMSE noise floors (as a percentage of the true displacement field amplitude) are plotted as a function of spatial period in Figure S13. We note that the x- and y-dimensions exhibit almost identical behaviors, whereas the z-dimension curves differ. We speculate that this may be a result of differing lateral versus axial resolutions of the microscope and/or the differing lateral versus axial side lengths of the volumetric images. We also note that the recovered displacement amplitude curve can report values that fall below the RMSE noise floor curve because, due to the averaging operation performed in Eqn. (D.3), the recovered displacement amplitude coefficient $U_{rec,(x/y/z),n}$ (derived from a spatial average of $\hat{u}_{(x/y/z),n}(x,y,z)$) has a much higher signal-to-noise ratio than the estimated displacement field $\hat{u}_{(x/y/z),n}(x,y,z)$ that it is derived from.

Using these curves, which are analogous to the modulation transfer function in optics, we define a 'cut-off spatial scale' at which *imregdemons* can track collagen deformations with reasonable performance. Since achieving quantitative traction force reconstructions in future experiments will rely on displacement data that exhibit both a high

accuracy and a high signal-to-noise ratio, we establish our ‘cut-off spatial scale’ based on those metrics. Using the *x*-/*y*-dimension curves (i.e., the ‘worst performing’ curves), we note that at a spatial period of 60 μm, the amplitude of the recovered displacement field falls to approximately 90% of the true displacement field amplitude. (That is, the algorithm begins to underestimate the true displacement field by >10% at spatial periods <60 μm.) Furthermore, at this same spatial scale, the recovered displacement field amplitude is approximately 10 times larger than the RMSE noise floor. Using these ‘amplitude recovery’ and ‘signal-to-noise-ratio’ criteria to define our cut-off for acceptable performance, we infer that the *imregdemons* function provides acceptable tracking of 5% strain down to a spatial scale of approximately 30 μm (~16 voxels). This 30 μm scale is one-half of the 60 μm spatial period we noted above, and corresponds to the size of each of the 2 distinct strain regimes contained in a single 60 μm ‘displacement wave’.

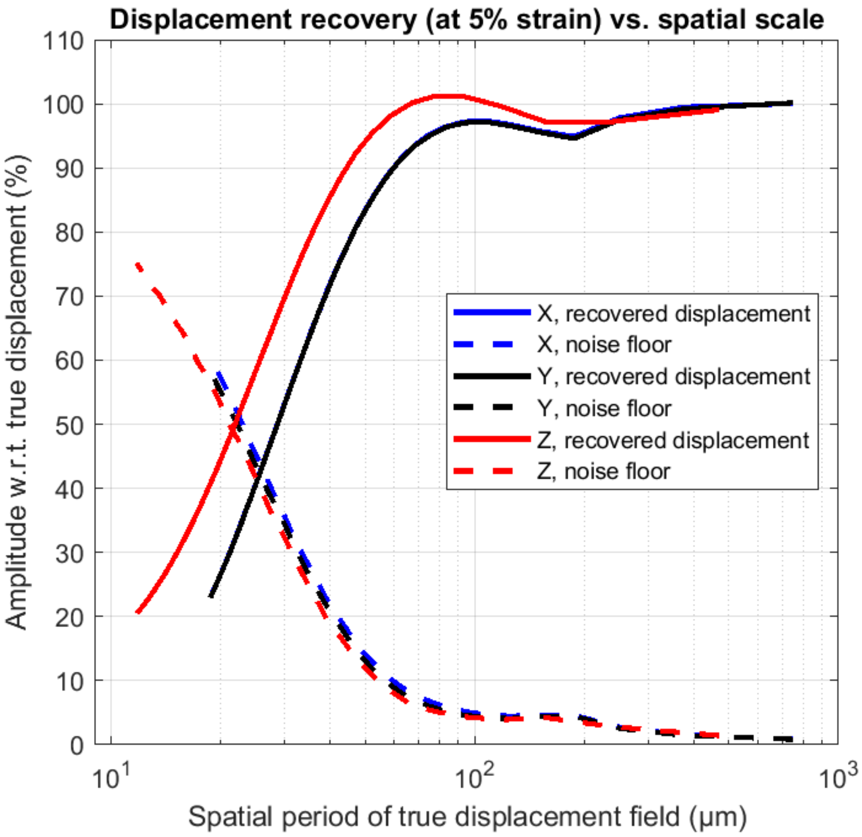

Figure S13. Recovered displacement amplitude and RMSE noise floor (as a percentage of the true applied displacement field amplitude) as a function of varying spatial scale. See text for details.

This 30 μm cut-off scale is substantially larger than the optical resolution of the imaging system (1.5 μm - 2.5 μm), and may be the result of multiple contributing factors. First, the input parameters that control the behavior of *imregdemons* (pyramid levels, iterations per pyramid level, and accumulated field smoothing) can all contribute to a low-pass filtering of the estimated displacement field. In particular, the algorithm first estimates displacements over large spatial scales (by analyzing low-resolution versions of the image data), and then works with progressively smaller spatial scales (eventually analyzing the image data at its full resolution).[10] An insufficient number of these ‘pyramid levels’ (and/or an insufficient number of iterations at each level) may cause a failure of the algorithm to fully converge to the desired solution. Failure to converge would be particularly impactful at small spatial scales, which are calculated primarily toward the end of the algorithm. Moreover, since the optical flow equations on which the Demons algorithm is based present an ill-posed system of equations, the algorithm must be regularized in order to obtain a solution.[8,9] In *imregdemons*, this is achieved via the ‘accumulated field smoothing’ parameter which controls a low-pass-filter-based regularization scheme.[10] The parameter must be present in order to avoid generating a very noisy solution, but this comes with the trade-off of poorer recovery and greater error at small spatial scales due to filtering.

We note that this 'regularization problem' emerges in other algorithms that can be used for fiber tracking. For example, cross-correlation-based tracking must select a 'window size' within which matching patterns will be identified. Larger windows reduce noise, but also reduce sensitivity to small-scale deformations.[11-13] Multi-scale approaches (analogous to the pyramid levels of *imregdemons*) can be used to help address this problem[14], but will ultimately reach a limit at some minimum window size).

Setting aside the specific effects of algorithm parameters, the structure of the collagen itself may impact performance. The presence of unique 'structures' (i.e., the specific patterns of light and dark that make up all images) play a role in displacement field recovery, since the Demons algorithm ultimately attempts to align 'matching patterns' within the reference and test images. Small-scale structures (such as collagen fibers) must be present throughout the volume in order to supply the small-scale features that enable the measurement of small-scale deformations. However, structures that are too small and/or too dense may exhibit reduced contrast (due to limitations of the imaging optics), and therefore degrade tracking performance. Our tissue-mimicking collagen samples provide a mixture of both small and large collagen fiber bundles (with the specific mixture emerging as a consequence of our collagen density and the temperature profile used during sample preparation; see the Methods). As a result, the performance that we report here is specific to not only the algorithm parameters that we have employed, but also to the properties of the samples that we have studied. Thus, any future studies that seek to expand upon our work with different parameters and/or samples will need to perform their own analyses, similar to that provided here.

Although our results do not provide a comprehensive picture of the performance of the *imregdemons* function in all scenarios (e.g., 'cut-off spatial scale' as a function of strain amplitude, collagen density, and/or algorithm parameters), these tests verify that the function can indeed track deformations in our collagen samples with displacement noise floors on the order of 0.05 voxels. However, as our above analysis shows, the *spatial scale* over which deformations are measured is an important factor to consider in addition to noise. For example, we demonstrated that our algorithm begins to fail at spatial scales smaller than 30 µm. As a consequence, although our technique is well-suited to measuring the deformations induced by spheroids, measuring small deformations induced by single cells may require more careful analysis and/or further developments. Our methods for assessing displacement and/or strain recovery at various spatial scales may be worth exploring in greater detail in the future. Such measurements may provide additional insight into the ability of our methods (or alternative techniques) to generate reliable displacement data that is suitable for the quantitative reconstruction of traction forces exerted by single cells and multicellular collectives.

Supplementary Note 6: Side-by-side comparison of temporal speckle contrast and confocal fluorescence

In order to verify the cellular content of our temporal speckle contrast images, a wild-type adipose stromal cell (WT ASC) spheroid was prepared and embedded in collagen as described in the Methods section of the main manuscript. After embedding, the spheroid was kept in an incubator (37ºC, 5% CO_2, 100% humidity) for 24 hours to allow for the formation of invasive strands (such as those seen in Supplementary Video 1). The spheroid was then removed from the incubator and immediately imaged on our OCT imaging system using the 'burst' imaging protocol described in the Methods.

After a single 'burst' of images was obtained, the sample was fixed with 4% paraformaldehyde (PFA) for 20 minutes and washed in 1× phosphate-buffered saline (PBS) three times. The sample was then permeabilized with 0.05% Triton X-100 (Alfa Aesar) in 1× PBS and blocked with 1% BSA (Thermo Fisher Scientific) in 1× PBS before incubation with Alexa Fluor 647 Phalloidin and 4',6-diamidino-2-phenylindole (DAPI) for one hour at room temperature. After this staining, the sample was washed three times with 1× PBS and stored in the dark at 4ºC prior to imaging. Confocal fluorescence images were obtained on a Zeiss LSM inverted i880 microscope using a W Plan-Apochromat 20×/1.0 Korr DIC M27 75 mm objective lens. The acquired confocal image stack had lateral dimensions of 708.5×708.5 μm^2 (512×512 voxels) and axial dimensions of 275 µm (137 voxels).

A temporal speckle contrast image stack (henceforth referred to as the 'OCT image stack', despite not being a typical OCT signal) was generated from the OCT 'burst' as described in the Methods. The fluorescence channels of the confocal image stack were merged into a single grayscale image stack. Maximum intensity projections of both image stacks were obtained along the vertical and horizontal dimensions to enable the manual identification of several dozen 'feature points' present in both image stacks. These feature points were then used to define an affine transformation (via least-squares fitting) that would map/register the confocal image stack onto the OCT image stack. We note that we specifically opted to use an affine transformation instead of a more 'flexible' method (such as an elastic transformation obtained via MATLAB's *imregdemons* function) in order to avoid artificially 'forcing' the confocal image stack to 'match' the OCT stack. Thus, we reduced the possibility of reporting a false, excessive correspondence between the two imaging modalities.

The confocal image stack was resampled (via the MATLAB function *interpn* using the 'spline' method) using this affine transformation, thereby yielding a confocal image stack registered to the OCT image stack. The OCT image stack was truncated along the axial dimension to remove depths not present/acquired in the confocal image stack. The two image stacks (confocal and truncated OCT) spanned a final axial range of 275 µm, beginning 100 µm within the sample beyond the coverslip base of the petri dish. Both image stacks were normalized by the 99th percentile of their respective (full-volume) histograms.

A depth-to-color projection image (analogous to those shown in Figures 1, 2, and 4 of the main manuscript) was generated for each volume. In the case of the confocal image stack, this was performed by multiplying the voxels at each depth by a unique RGB value, followed by a maximum intensity projection of each color channel along depth. This is identical to the operation used to perform the depth-to-color projection of the temporal speckle contrast images in Figures 1, 2, and 4 of the main manuscript. However, in order to allow for easier qualitative visual comparison, a modified depth-to-color projection was used for the OCT image stack here, which we will explain below.

In the confocal image stack, the fluorescence signal decays very quickly upon entering the spheroid body (see the top panel of Figure S14). As a result, significant fluorescence signals return only from cells near the spheroid-collagen interface. Thus, in the depth-to-color projection image (top-right panel of Figure S15), we effectively observe a color-coded contour plot of the spheroid surface. The blue regions correspond to those surfaces which lie closest to the microscope objective, whereas the regions that span the spectrum from blue to red denote regions at progressively deeper locations within the sample.

This same general trend can be seen in the invasive branches depicted in Figures 1, 2, and 4 of the main manuscript. Note, however, that the 'core' of the spheroid body in the depth-to-color projection of Figure 1 appears nearly white. This is because the temporal speckle contrast images generated via OCT obtain signals both at the spheroid-collagen interface and very deep within the spheroid. (For example, see the bottom panel of Figure S14.) Thus, strong 'blues'

from the surface of the spheroid 'core' are superimposed with strong 'reds' from deeper inside the spheroid (and every depth/color in between) to produce a 'white' image following the maximum intensity projection performed on each color channel (i.e., structures occurring at different depths are indiscriminately 'smashed' together in those images.)

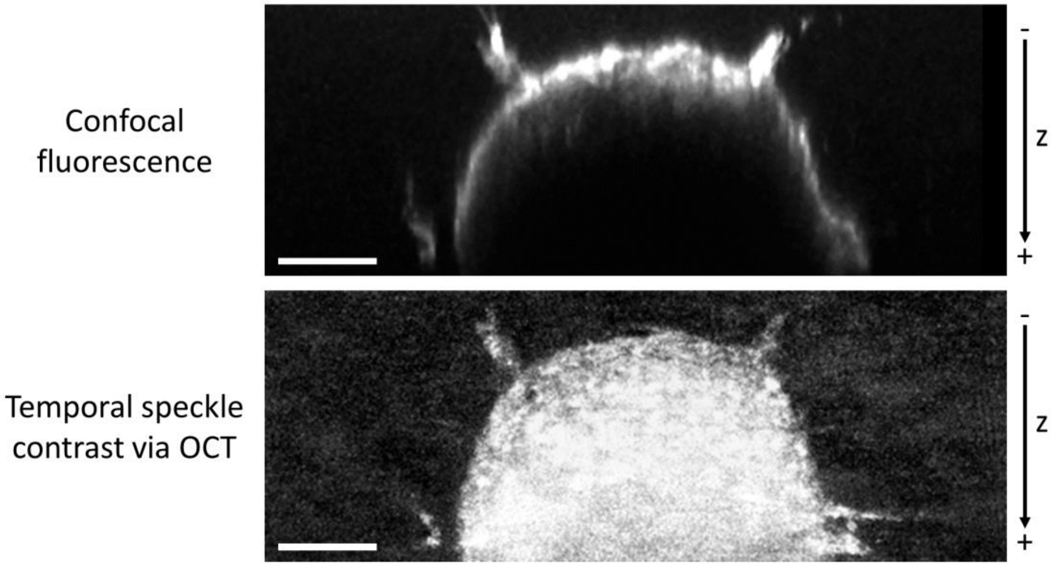

Figure S14. Comparison of signal decay with depth in confocal fluorescence (top) versus temporal speckle contrast via OCT (bottom). Scale bar = 100 µm. See text for details.

Here, for the purposes of visual comparison, we modified the depth-to-color projection for the OCT image stack so that it mimics the appearance of the depth-to-color projection of the confocal image stack. Specifically, this modification mimics the rapid 'decay' exhibited by the fluorescence signals of the confocal image stack. Denoting the temporal speckle contrast image signal by $I(x, y, z)$, an 'attenuated signal' was generated via:

$$I_{\alpha}(x, y, z) = I(x, y, z) \exp\left(-\alpha \int_0^z I(x, y, z')\, \mathrm{d}z'\right), \tag{E.1}$$

where a decay constant of $\alpha = 0.07$ was (manually) chosen to achieve the desired 'decay' behavior. The resulting 'attenuated' temporal speckle contrast image $I_{\alpha}(x, y, z)$ was then subjected to the previously described depth-to-color projection operation, yielding the image in the top-left panel of Figure S15. Overall, this reveals an excellent visual correspondence between our label-free temporal speckle contrast method and standard confocal fluorescence.

Next, the image stacks were binarized via single-level thresholding, followed by volume-filtering to remove small objects. These binary image stacks were then subjected to a depth-to-color projection (in which the color of the first non-zero voxel along the depth dimension was retained), yielding the images in the bottom row of Figure S15. These images provide a better sense of the 3D structures present in the two images (without depth-dependent variations in intensity or overlap of signals from multiple depths). Again, we observe an excellent visual correspondence between the two images. However, we do note discrepancies between the two images when examining fine protrusions/structures.

In order to quantify these discrepancies, first, a spherical mask was defined to denote the voxels the make up the spheroid 'core'. Since the signal decay behavior of the confocal and OCT image stacks differ drastically within the core region, we defined the spherical mask in order to exclude this region from our subsequent analysis. Next, taking the binarized confocal image stack to represent the 'ground truth' indicator of cells (i.e., whether or not cells are present in a given voxel), and the binarized OCT stack to represent a 'test' indicator for cells (i.e., whether or not cells are reported in a given voxel), we computed a 2×2 contingency table to record the total number of voxels (outside the spheroid core) corresponding to each of the following categories: true positive (TP, a cell was reported and present),

false positive (FP, a cell was reported but not present), false negative (FN, a cell was not reported but was present), and true negative (TN, no cell was reported or present). Specifically, denoting voxel *i* of the binarized OCT stack and confocal stack by A_i and B_i, respectively, and denoting the set of voxels outside the spheroid core (i.e., the set of voxels *not* within the spherical mask) by the set C, we define:

$$TP = \sum_{i \in C} A_i B_i, \quad FP = \sum_{i \in C} A_i (1 - B_i), \quad FN = \sum_{i \in C} (1 - A_i) B_i, \quad TN = \sum_{i \in C} (1 - A_i)(1 - B_i) \qquad \text{(E.2)}$$

where $A_i \in \{0,1\}$ denotes whether cells are $\{\text{not reported, reported}\}$, $B_i \in \{0,1\}$ denotes whether cells are $\{\text{not present, present}\}$, multiplication is equivalent to a logical 'AND' operation, subtraction from 1 is equivalent to a logical 'NOT' operation, and summation is equivalent to counting the number of voxels that satisfy a given condition. (For example, $\sum_{i \in C} A_i (1 - B_i)$ can be interpreted as "Count the number of voxels outside the spheroid core for which a cell was reported AND a cell was NOT present", i.e., the false positive voxel count.) The resulting values obtained were: TP=42,692 voxels, FP=26,581 voxels, FN=82,024 voxels, TN=18,739,804 voxels. These results are summarized in Figure S16 and discussed below.

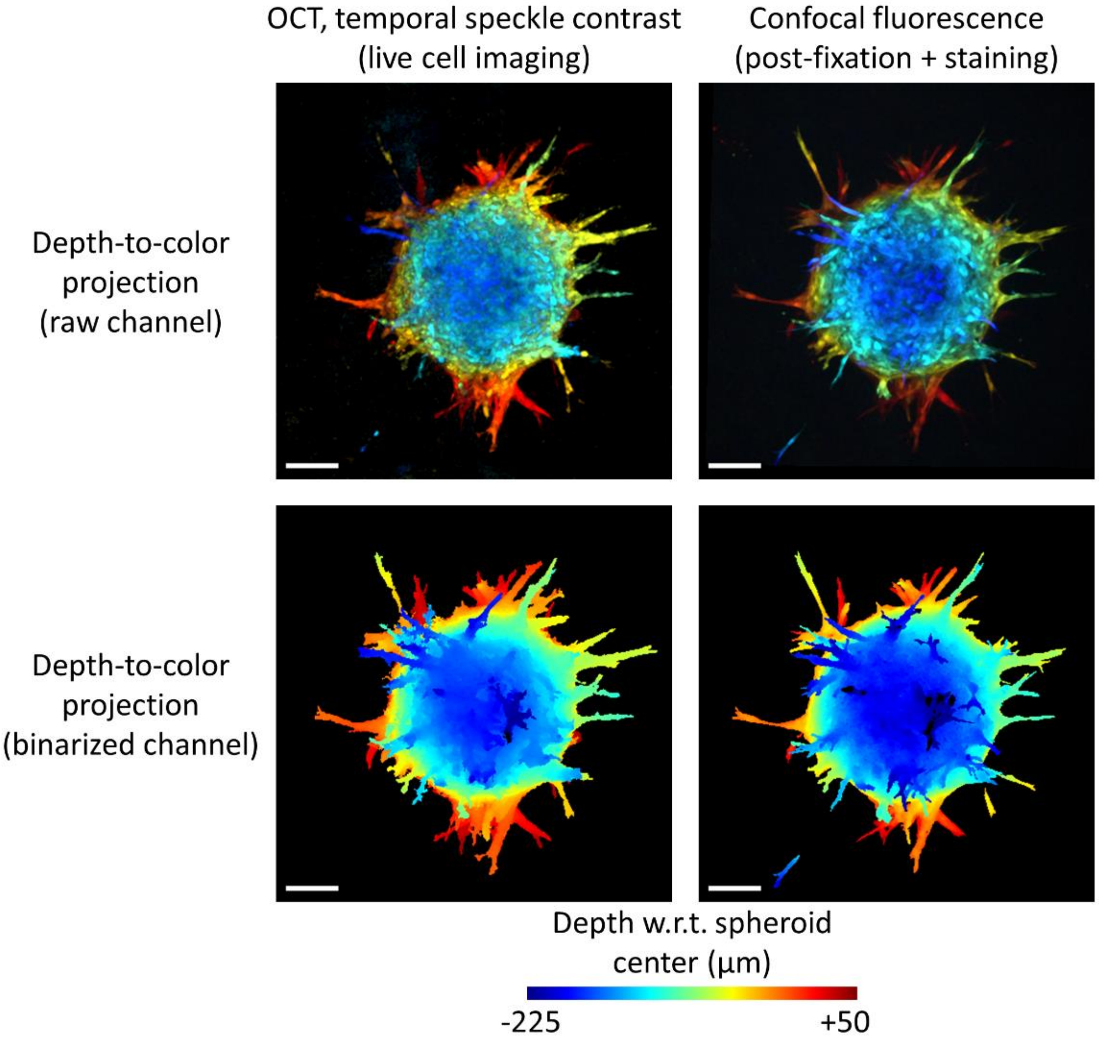

Figure S15. Invasion of a WT ASC monoculture spheroid into the surrounding collagen substrate, as visualized via our label-free temporal speckle contrast method (left) and standard confocal fluorescence imaging (right). Scale bar = 100 μm. See text for details.

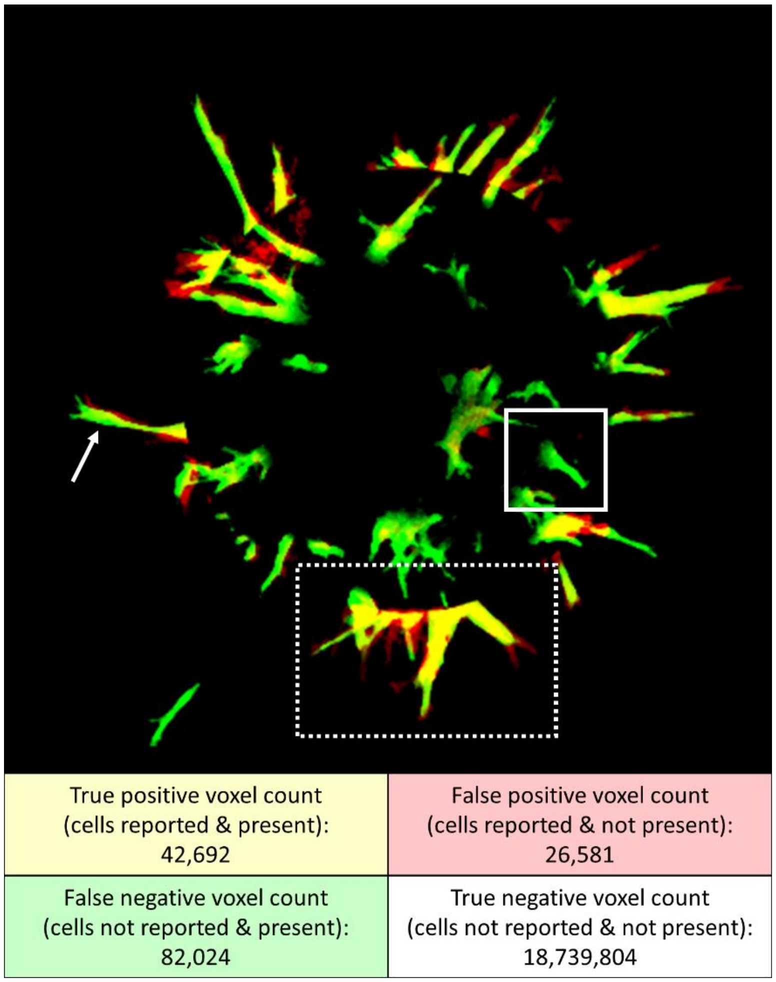

True positive voxel count (cells reported & present): 42,692	False positive voxel count (cells reported & not present): 26,581
False negative voxel count (cells not reported & present): 82,024	True negative voxel count (cells not reported & not present): 18,739,804

Figure S16. Summary of correspondence between the binarized temporal speckle contrast channel (red) and the binarized confocal fluorescence channel (green) of the spheroid from Figures S14 and S15. See text for details.

The image panel in Figure S16 represents a summation projection of both channels (temporal speckle contrast in red, and confocal fluorescence in green) along the axial dimension, following the application of the previously defined spherical mask. Thus, the visible structures represent invasive protrusions or other structures emerging from the spheroid core. Regions containing exclusively red or green voxels correspond to regions that contributed to our FP or FN voxel counts, respectively. Regions of channel overlap (yellow) can correspond to TP regions, but may also contain FP/FN regions due to the summation projection used to produce the image. TN voxels and excluded voxels (from the spheroid core) did not contribute to the formation of the image panel. (Note that all counts were obtained from the original volumetric data. The image panel in Figure S16 is merely for visualization purposes.) Using this visualization, we can observe the correspondence of many structures in both imaging channels. However, fine structural details can differ. For example, consider the following regions: 1) the lower region outlined by the dotted rectangle, where fine protrusions are visible in the temporal speckle contrast channel, but not the confocal fluorescence channel, 2) the region outlined by the solid rectangle, where structures are visible in the confocal fluorescence channel, but not the temporal speckle contrast channel, and 3) the protrusion indicated by the arrow, for which the two channels appear to be imperfectly registered.

Overall, these structural features reveal that our contingency table cannot be used to provide a definitive quantitative analysis of the correspondence between the two imaging modalities. In general, since these images were acquired at different times (pre- and post-fixation) on different microscope systems, differences in the optical PSFs, errors in the digital image registration procedure, and/or actual structural differences between the images (due to the time elapsed between OCT imaging and sample fixation and/or structural changes resulting from fixation) confound the generation

and interpretation of quantitative metrics. We believe these factors to be the most plausible explanation for the high FP/FN counts observed relative to the TP counts, despite the excellent qualitative correspondence that can be observed in Figure S15. An imaging system that enables simultaneous, optically co-registered OCT and confocal fluorescence imaging would be an ideal system for performing a robust comparison of temporal speckle contrast versus fluorescent labeling for identifying cellular structures. However, dual-modality OCT-confocal microscopes suitable for traction force microscopy applications are a rare-to-nonexistent commodity, to the best of our knowledge. Thus, we have plans to develop and use such an imaging system in our future research. A high-resolution OCT-confocal fluorescence microscope would not only enable a rigorous validation of the preliminary comparisons detailed above, the two modalities may provide synergies for more general research purposes, such as utilizing OCT to perform rapid and long-term imaging of spheroid/collagen features while performing intermittent confocal fluorescence imaging to leverage label specificity for various applications. In the present, we believe that our results shown here (e.g., in Fig. S15) give us a strong indication that our temporal speckle contrast method is a viable technique for generating label-free images of invasive spheroids embedded in scattering collagen substrates. Future investigations using co-registered OCT-confocal fluorescence microscopes will likely be valuable for providing robust quantification of the strengths and weaknesses of our technique.

Supplementary Video Captions

Supplementary Video 1: Animation of the spheroid depicted in Figure 1. Panels (top-left, top-middle, top-right, bottom-left, and bottom-middle) correspond to rows 1-5 from Figure 1, respectively.

Supplementary Video 2: Animation of the spheroid depicted in Figure 2. Panels (top-left, top-middle, top-right, bottom-left, and bottom-middle) correspond to rows 1-5 from Figure 2, respectively.

Supplementary Video 3: Animation of the panels depicted in Figure 3. The left-hand side depicts panels with only scattering contrast. The right-hand side depicts the same panels including superimposed temporal speckle contrast-based labeling in green (i.e., as shown in Figure 3).

Supplementary Video 4: Animation of Figure 4. Time-stamps shown are in HH:MM format.

Supplementary references

1 Mulligan, J. A., Feng, X. & Adie, S. G. Quantitative reconstruction of time-varying 3D cell forces with traction force optical coherence microscopy. *Sci Rep* **9**, 4086, doi:10.1038/s41598-019-40608-4 (2019).

2 Chelliyil, R. G., Ralston, T. S., Marks, D. L. & Boppart, S. A. High-speed processing architecture for spectral-domain optical coherence microscopy. *J Biomed Opt* **13**, 044013, doi:10.1117/1.2960018 (2008).

3 Liu, X. & Kang, J. U. Compressize SD-OCT: the application of compressed sensing in spectral domain optical coherence tomography. *Opt Express* **18**, 22010-22019 (2010).

4 Rivet, S. *et al.* Complex master slave interferometry. *Opt Express* **24**, 2885-2904, doi:10.1364/OE.24.002885 (2016).

5 Kumar, A. *et al.* Anisotropic aberration correction using region of interest based digital adaptive optics in Fourier domain OCT. *Biomedical Optics Express* **6**, 1124-1134, doi:10.1364/Boe.6.001124 (2015).

6 Kumar, A., Drexler, W. & Leitgeb, R. A. Subaperture correlation based digital adaptive optics for full field optical coherence tomography. *Opt Express* **21**, 10850-10866, doi:10.1364/OE.21.010850 (2013).

7 Adie, S. G., Graf, B. W., Ahmad, A., Carney, P. S. & Boppart, S. A. Computational adaptive optics for broadband optical interferometric tomography of biological tissue. *Proc Natl Acad Sci U S A* **109**, 7175-7180, doi:10.1073/pnas.1121193109 (2012).

8 Pennec, X., Cachier, P. & Ayache, N. Understanding the "Demon's Algorithm": 3D non-rigid registration by gradient descent. *Lect Notes Comput Sc* **1679**, 597-605 (1999).

9 Vercauteren, T., Pennec, X., Perchant, A. & Ayache, N. Diffeomorphic demons: efficient non-parametric image registration. *Neuroimage* **45**, S61-72, doi:10.1016/j.neuroimage.2008.10.040 (2009).

10 *imregdemons*, <https://www.mathworks.com/help/images/ref/imregdemons.html> (2020).

11 Kim, J., Jones, C. A., Groves, N. S. & Sun, B. Three-Dimensional Reflectance Traction Microscopy. *PLoS One* **11**, e0156797, doi:10.1371/journal.pone.0156797 (2016).

12 Mulligan, J. A., Bordeleau, F., Reinhart-King, C. A. & Adie, S. G. Measurement of dynamic cell-induced 3D displacement fields in vitro for traction force optical coherence microscopy. *Biomed. Opt. Express* **8**, 1152-1171, doi:10.1364/BOE.8.001152 (2017).

13 Mulligan, J. A., Bordeleau, F., Reinhart-King, C. A. & Adie, S. G. in *Biomechanics in Oncology* (eds C. Dong, N. Kuhn, & K. Konstantopoulos) (Springer Nature Switzerland AG, 2018).

14 Bar-Kochba, E., Toyjanova, J., Andrews, E., Kim, K. S. & Franck, C. A fast iterative digital volume correlation algorithm for large deformations. *Exp. Mech.* **55**, 261-274, doi:10.1007/s11340-014-9874-2 (2015).
